# Facile Fabrication of High-Performance Thin Film Nanocomposite Desalination Membranes Imbedded with Alkyl Group-Capped Silica Nanoparticles

**DOI:** 10.3390/polym12061415

**Published:** 2020-06-24

**Authors:** Biqin Wu, Shuhao Wang, Jian Wang, Xiaoxiao Song, Yong Zhou, Congjie Gao

**Affiliations:** 1 Center for Membrane Separation and Water Science & Technology, Ocean College, Zhejiang University of Technology, Hangzhou 310014, China; Peachy_Wu@163.com (B.W.); shuhao100e@126.com (S.W.); zhouy@zjut.edu.cn (Y.Z.); gaocj@zjut.edu.cn (C.G.); 2 Institute of Tianjin Seawater Desalination and Multipurpose Utilization, Ministry of Natural Resources, Tianjin 300192, China; wangjian_isdmu@163.com

**Keywords:** TFN-RO membrane, alkyl capped silica nanoparticles, confined growth, interfacial polymerisation, desalination

## Abstract

The advantages of thin film nanocomposite reverse osmosis (TFN-RO) membranes have been demonstrated by numerous studies within the last decade. This study proposes a facile and novel method to tune the microscale and nanoscale structures, which has good potential to fabricate high-performance TFN-RO membranes. This method involves the addition of alkyl capped silica nanoparticles (alkyl-silica NPs) into the organic phase during interfacial polymerization (IP). We discovered for the first time that the high concentration alkyl-silica NPs in organic solvent isopar-G can limit the diffusion of MPD molecules at the interface, therefore shaping the intrinsic thickness and microstructures of the PA layer. Moreover, the alkyl group modification greatly reduces the NPs agglomeration and increases the compatibility between the NPs and the PA matrix. We further demonstrate that the doping of alkyl-silica NPs impacts the performance of the TFN-RO membrane by affecting intrinsic thickness, higher surface area, hydrophobic plugging effect, and higher surface charge by a series of characterization. At brackish water desalination conditions (2000 ppm NaCl, 1.55 MPa), the optimal brackish water flux was 55.3 L/m^2^∙h, and the rejection was maintained at 99.6%, or even exceeded this baseline. At seawater desalination conditions (32,000 ppm NaCl, 5.5 MPa), the optimized seawater flux reached 67.7 L/m^2^∙h, and the rejection was sustained at 99.4%. Moreover, the boron rejection was elevated by 11%, which benefits from a hydrophobic plugging effect of the alkyl groups.

## 1. Introduction

Owing to its low energy consumption and efficiency, reverse osmosis (RO) has become the most popular purification methods to provide for clean water needs through membrane-based processes such as desalination, water reclamation, and water detoxification, etc. [1,2,3,4]. The RO membrane technology still faces the problems of membrane fouling and scaling in the practical application, which seriously lower membrane performance and restricts further development [5,6,7]. The recently established polyamide (PA)-based thin film nanocomposite reverse osmosis (TFN-RO) membranes have proven to be promising by both research and industrial efforts over the past decade [8,9,10,11,12,13,14]. The performance of the TFN-RO membranes is significantly impacted by the nanoparticles (NPs) blended into the PA polymer matrix, such as their intrinsic porosity, hydrophilicity, and interfacial porosity, etc. Among various blending methods, the in situ blending of the NPs with the aqueous solution or the organic solution during the interfacial polymerization of the PA nanofilm is most frequently used due to its high efficiency, ease manipulation, controllable loading amount and readily scalability.

To date, most thin film nanocomposite (TFN) membranes have been prepared by blending hydrophilic nanoparticles (NPs) with the amine aqueous phase during the interfacial polymerization (IP) step due to the facile synthesis of the nanomaterials and ease of fabrication process. However, Blending NPs with an amine aqueous solution phase faces several major issues such as low blending efficiency, the tendency of generating defects, and long-term instability. On one hand, the amine monomers need to diffuse into the organic phase to react with acyl chloride monomer. Meanwhile, hydrophilic NPs can hardly diffuse together with the amine molecules due to their much larger molecular weight and immiscibility into the organic phase, but rather remain between the polysulfone (PSF) supporting layer and the PA layer [4,15,16,17,18]. Moreover, the incorporation of hydrophilic NPs, such as carbon nanotubes [19,20], TiO_2_ [21], and silver NPs (AgNPs) [4,22], results in defects in the PA layer. For example, nanosized gaps were observed around imbedded hydrophilic AgNPs within the PA layer, therefore forming defects at the interface of NPs and polyamide. In this case, the incorporation of inorganic NPs allows for the enhancement of surface hydrophilicity and therefore higher water flux, but meanwhile introduces ion-nonselective voids, which negatively impacts the salt rejection [22]. Furthermore, due to the weak interactions between the NPs and membrane, the NPs are prone to be washed off from the membrane under higher pressure, resulting in the secondary pollution of the water source [23].

Alternatively, when NPs are added to the organic phase, the properties of the PA separation layer are enhanced and membranes contain fewer defects, resulting in higher roughness and improved water flux while maintaining salt rejection [9,24]. For example, Hoek et al. prepared high-rejection TFN membranes using the organic solution introduced zeolites. However, the preparation, activation and dispersion of the zeolites are time and cost consuming [8]. Wang et al. added different sizes of ZIF-8 into the oil phase to produce a series of TFN RO membranes. The salt rejection could be maintained at around 99% [25]. Shen et al. doped the silicon tetrachloride, a silica precursor, into the acyl chloride solution to carry out IP with amine to form RO membrane. A significantly higher permeability was obtained, but the rejection was lower than 97% at brackish water conditions [26]. Up to now, facile methods to fabricate high-rejection TFN desalination membranes with low-cost nanomaterials have rarely been reported. In this work, we report a facile method to prepare high-performance TFN-RO membrane for seawater desalination purposes using commercially available, fumed silica NPs, which are modified by silicon alkyl groups and readily dissolvable in the isopar-G solvent to form transparent organic solutions. Due to the organophilic properties of the alkyl groups, they have better opportunities to insert into the PA chains [27], forging compatibility between the NPs and the PA network. Thus, the hydrophobic NPs will be immobilized in the PA layer and imbedded into the polymer matrix. Moreover, due to the ultrafine size of the NPs (~10 nm), they could be better imbedded with the PA matrix, rendering better compatibility between the NPs and PA network. This work examines the high concentration NPs added in the organic phase and for the first time analyzes their shaping effect on the nanoscale and separation properties of the resultant TFN membrane. The performance of the resultant TFN membrane is examined in both brackish water and seawater desalination conditions.

## 2. Experiments and Methods

### 2.1. Materials and Reagents

Polysulfone (PSF) UF membranes with a MWCO of 35 kDa (laboratory pilot line) were used as the substrate membrane [28]. Alkyl-silica NPs including AEROSIL^®^ R 812 S, AEROSIL^®^ R 972 and AEROSIL^®^ R 974 were purchased from Evonik Resource Efficiency GmbH. M-phenylene diamine (MPD, 99%), camphorsulfonic acid (CSA, 99%), 1,3,5-benzenetricarbonyl trichloride (TMC, 98%), boric acid and triethylamine (TEA) were purchased from Aladdin. Sodium chloride (NaCl) was purchased from Guangdong Guanghua Sci-Tech CO., LTD., Shantou, China and sodium hydroxide (NaOH) was from Xilong Scientific Co., LTD., Shantou, China. Isopar-G was obtained from ExxonMobil Chemical Company, Houston, TX, USA while dimethylformamide (DMF) was from Wuxi Haishuo Biological CO., LTD., Wuxi, China. All reagents were analytical grade unless otherwise stated.

### 2.2. Membrane Synthesis

**Figure 1:**
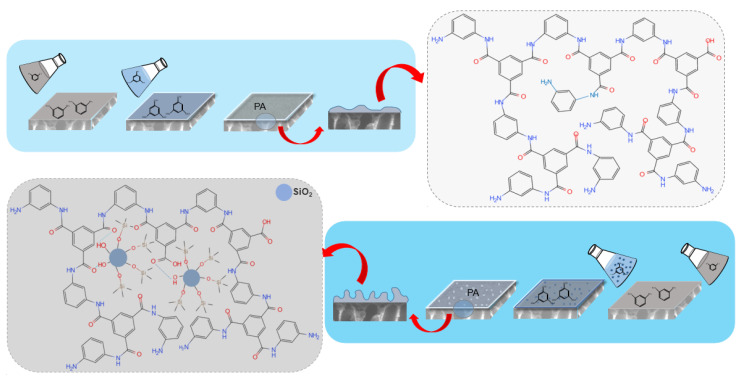
Fabrication progress and mechanism diagram for the TFC (**top**) and TFN (**bottom**) RO membranes. In the TFN-RO membrane, a significant amount of alkyl-silica NPs was imbedded in the membrane; hydrogen bonds were formed between the hydroxyl groups and amide-bonded oxygens while other branch chains of the alkyl-silica NPs were intertwined with the polymer matrix.

The fabrication process of the RO membranes is illustrated in Figure 1. The TFC membrane was fabricated by IP process, wherein a 2% (*w*/*v*) MPD aqueous phase (pH was adjusted to 9.5 using a CSA-TEA buffer solution) reacted with a 0.1% (*w*/*v*) TMC dissolved in isopar-G organic solution. The reaction was carried out on a PSF ultrafiltration porous substrate clamped between two Teflon plate frames. First of all, the substrate was soaked in the MPD solution for 2 min, the residual solution on the substrate membrane was removed and the surface was slightly dried with sweeping N_2_. Secondly, the above membrane was immersed in the TMC solution for 1 min and removed the excess organic phase, followed by drying to form an original thin layer. It was then heated in an oven at 95 °C for 5 min to form a dense layer of PA.

The TFN membranes were prepared using the same procedure with alkyl-silica NPs blended in the organic phase. Specifically, a series of concentrations (0.02%, 0.05%, 0.1%, 0.2% and 0.5% (*w*/*v*)) of NPs were prepared by dissolving NPs in the organic phase, followed by ultrasonication for 1 h prior to the IP process. The intrinsic parameters of the three NPs used in this study, R812S, R974, and R972, are shown in Table 1. To facilitate discussion, the TFN membranes are designated a short name with the NPs type and concentration. For example, TFN membrane fabricated using 0.1% (*w*/*v*) R812S NPs is designated with the name of TFN-812S-0.1, etc.

### 2.3. Membrane Characterization

The fabricated membranes were cleaned with deionized water and dried in a vacuum oven at room temperature for 24 h before any characterization was conducted.

#### 2.3.1. Membrane Morphology

The top and bottom surfaces and the cross-section of each membrane were examined by scanning electron microscope (SEM, SU8010, HITACHI, Tokyo, Japan) at 10 kV. To characterize the PA on the back surface, DMF was used to dissolve the PSF supporting layer. The isolated PA layers, loaded on a silicon wafer (0.5 × 0.5 cm^2^), were dried in a vacuum drier for at least 24 h prior to characterization [29]. To observe the morphology and layer thickness in cross-sections, the samples were frozen in liquid nitrogen and then fractured. Before observation, all samples were coated with gold for 45 s. Transmission electron microscopy (TEM, JEM-1200EX/JEM-1230, JOEL, Tokyo, Japan) was conducted at 80 kV to examine the top surfaces and cross-sections for evaluating the size distributions of the alkyl-silica NPs within the membranes. Briefly, after dissolving the PSF substrate membrane using DMF, the PA membranes were transferred to copper grid carriers for TEM observation. The cross-sectional membrane samples were embedded in resin for 8 h and then cut into approximately 80-nm-thick sections using a microtome and then transferred onto copper grids. Atomic force microscopy (AFM, Bruker, Dimension Icon, Billerica, MA, USA) was used to observe the surface roughness of each 5 × 5 μm^2^ membrane by comparing *Ra* and *Rq* values from the obtained 3D morphology diagram.

#### 2.3.2. Chemical Composition

To verify the alkyl-silica NPs doping and evaluate the dispersion of the NPs on the membranes, TEM and dynamic light scattering (DLS, Omni, Holtsville, NY, USA) were used to measure the sizes of the alkyl-silica NPs in the membrane and in isopar-G to evaluate their dispersibility in the solvent.

A small angle x-ray scattering spectrometer (SAXS, Anton Paar, Graz, Austria) was used to compare the TFC and TFN membranes and to describe the structure variation due to alkyl-silica NPs doping in the PA rejection layer [30,31,32]. As described above, DMF was used to dissolve the PSF supporting layer and to isolate the PA layer. To facilitate observation, 5 PA layers were stacked layer-by-layer on a silicon wafer (0.5 × 0.5 cm^2^).

Fourier-transform infrared (FT-IR, Thermo Fisher Nicolet-is50, Shanghai, China) spectroscopy was used to analyze the functional groups of the different alkyl-silica NPs. The NPs were first mixed with KBr and then compressed into a semi-transparent pellet to measure the transmittance at room temperature over an infrared range from 400 to 4000 cm^−1^ [33]. Detailed information was obtained from the attenuated total reflection Fourier-transform infrared (ATR-FTIR) signal and analyzed to identify the functional groups on the membrane surfaces. The measurements were conducted at room temperature using air as the background.

#### 2.3.3. Interfacial Properties

The hydrophilicity/hydrophobicity of each membrane surface was measured with a contact angle meter (CA, OCA15EC, Dataphysics, Filderstadt, Germany) using the sessile drop technique with deionized (DI) water as the reference liquid. A droplet of DI water of approximately 3 μL was deposited on the leveled membrane surface to measure the contact angle of each sample. The mean static contact angle was calculated from six different positions.

A solid-surface Zeta potentiometer (Zeta potential, Anton Paar SurPASS 3, Austria) was used to characterize the charges on the membrane surface. The background electrolyte solution was 1 mmol/L KCl, 0.05 mol/L HCl and 0.05 mol/L NaOH were used to adjust the pH from 10 to 3.

### 2.4. Separation Performance Testing

**Figure 2:**
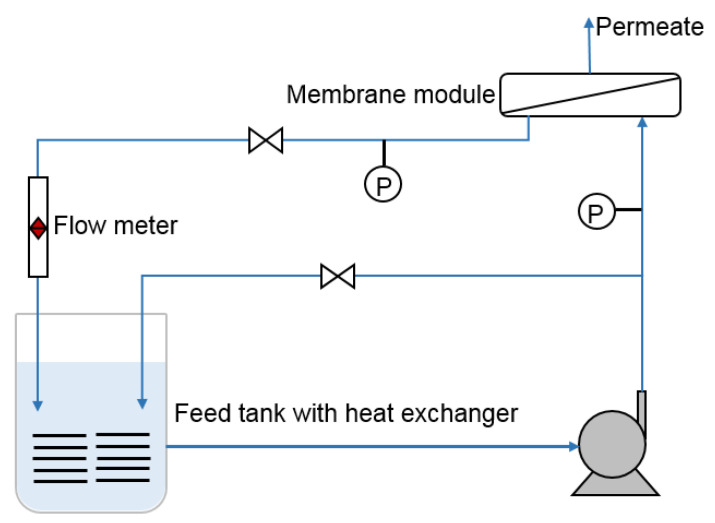
Schematic diagram of the experimental apparatus used to evaluate the permselectivity of the fabricated RO membranes.

The membrane separation performances (flux and rejection) were evaluated in a high-pressure cross-flow RO evaluation setup (Figure 2). Before the experiment, the device was operated for 1 h to ensure that the system pressure was stable. First, pure water was run for 1 h to compute the *A* value. This one-hour flux and salt rejection of simulated brackish water (2000 ppm NaCl) were used to estimate the *B* value. The discrepant test pressure was 16 bar for pure and brackish water and 55 bar for seawater. The other test conditions were constant (pH = 8 ± 0.2 and T = 25 ± 0.5 °C).

The permeate water flux, *J* (L/m^2^∙h), was calculated according to the equation *J* = V/(A × Δt), where V is the volume of the permeated solution (L), A is the effective membrane area (19.63 cm^2^) and Δt (1 h) is the permeation time of the experiment. The pure water permeability coefficient, *A* value (L/m^2^∙h∙bar), was calculated according to the equation *A* = *J*/ΔP, where ΔP is the operating pressure (bar) [22]. The rejection *R* (%) was calculated using the equation *R* = (1 − C_p_/C_f_) × 100%, where C_p_ and C_f_ denote the concentrations of the permeation solution and feed solution, respectively. The salt permeability coefficient, *B* value (L/m^2^∙h), was calculated using the equation *B* = *J* (1 − *R*)/*R*. To further analyze the transport process of boron, the permeability coefficient, *Bs*, of boron was calculated using the equation *Js* = *Bs* × (C_f,B_ − C_p,B_), where *Js* is the permeation flux of boron, C_f,B_ and C_p,B_ represent the concentrations of the feed solution and permeate solution, respectively [33,34]. The NaCl concentration was evaluated based on the conductivity, whereas the boron concentration was measured using an inductively coupled plasma-optical emission spectrometer (ICP-OES, PerkinElmer, AvioTM 200, Shanghai, China).

## 3. Results and Discussion

### 3.1. Properties of the Alkyl-Silica NPs

**Figure 3:**
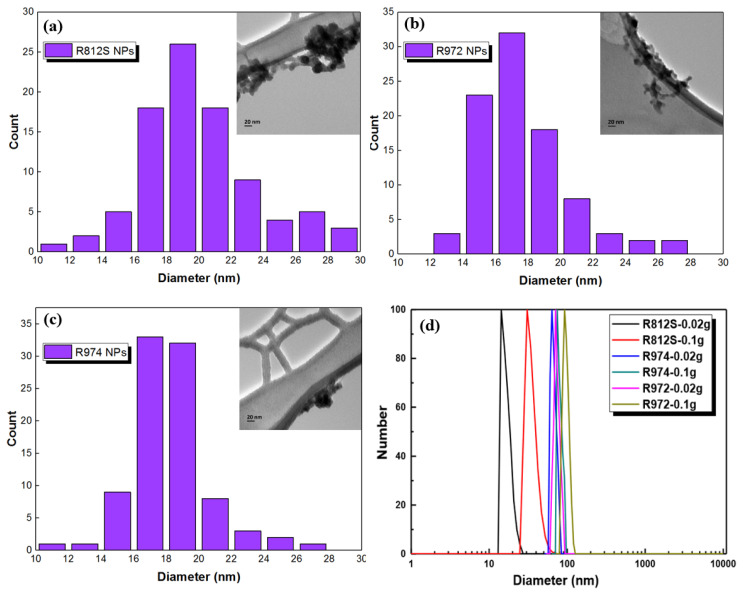
(**a**–**c**) Number of NPs per size and TEM images (inserted) of the three types of alkyl-silica NPs, the size distribution of each NP was determined based on 100 NPs of each type; (**d**) diameters of the NPs at different concentrations in the isopar-G solvent as measured by DLS.

The alkyl-silica NPs can be well dispersed fully in isopar-G solvent and form a clear solution. Even when the particle mass fraction was increased to 5% (*w*/*v*), the solution remained transparent. The 0.5% NPs dispersion in isopar-G was stable in ambient conditions for more than 1 month. The TEM images of the three silica NPs are presented in Figure 3a–d, which shows the size of the alkyl-silica NPs of narrow distributions around 20 nm. It is worth mentioning that although the particle size distribution did not show much variation among these NPs. The difference in BET surface area (Table 1, R812S > R974 > R972) of the NPs may be explained by the different dispersity of the NPs, modified by different alkyl groups/different procedures that modified the NPs, which follows the order of R812S > R974 > R972. DLS results indicate that the R812S is dispersed very well at the concentration of 0.02% (~20 nm) but became aggregated at the concentration of 0.1% (~40 nm). Besides, at the same concentration, the aggregates of the NPs follow the order of R812S < R974 < R972, this agrees well with the dispersity order of the three NPs.

### 3.2. Morphologies of the Fabricated Membranes

**Figure 4:**
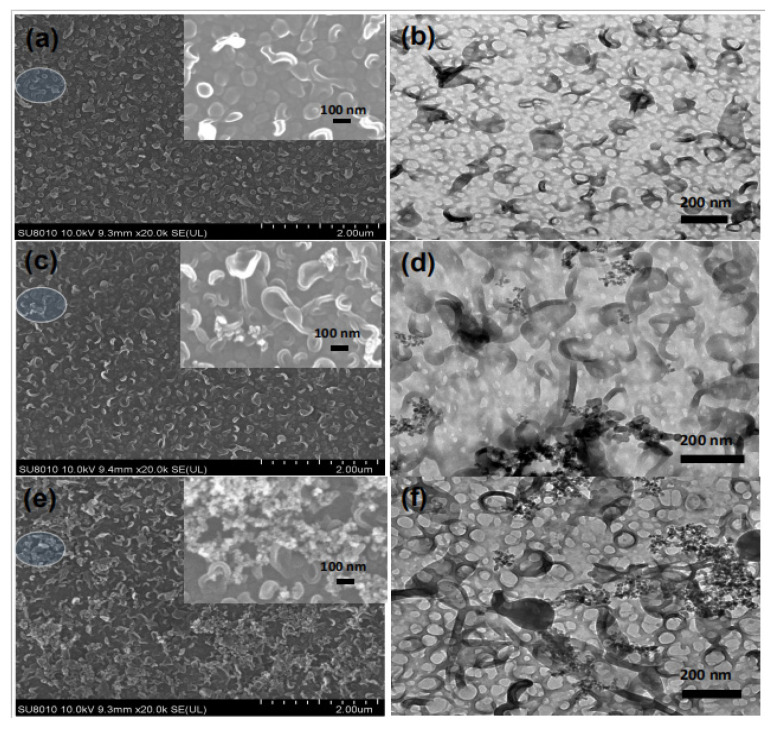
SEM (**left**) and TEM (**right**) images of membranes with different concentrations of R812S NPs: (**a**,**b**) no NPs, (**c**,**d**) 0.02% (*w*/*v*), (**e**,**f**) and 0.1% (*w*/*v*).

**Figure 5:**
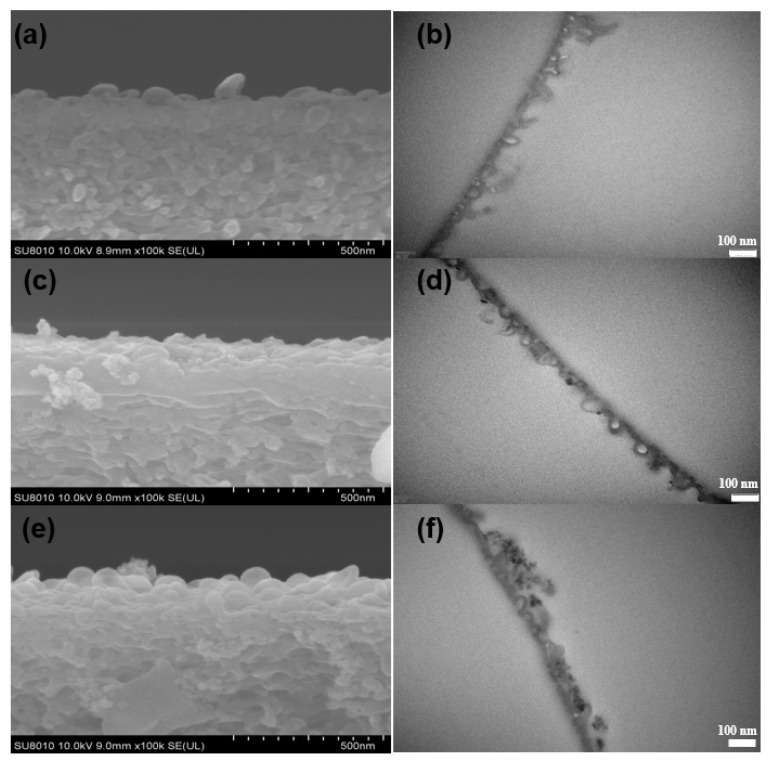
SEM (**left**) and TEM (**right**) images of cross-sections of membranes with different concentrations of R812S NPs: (**a**,**b**) no NPs, (**c**,**d**) 0.02% (*w*/*v*) and (**e**,**f**) 0.1% (*w*/*v*).

**Figure 6:**
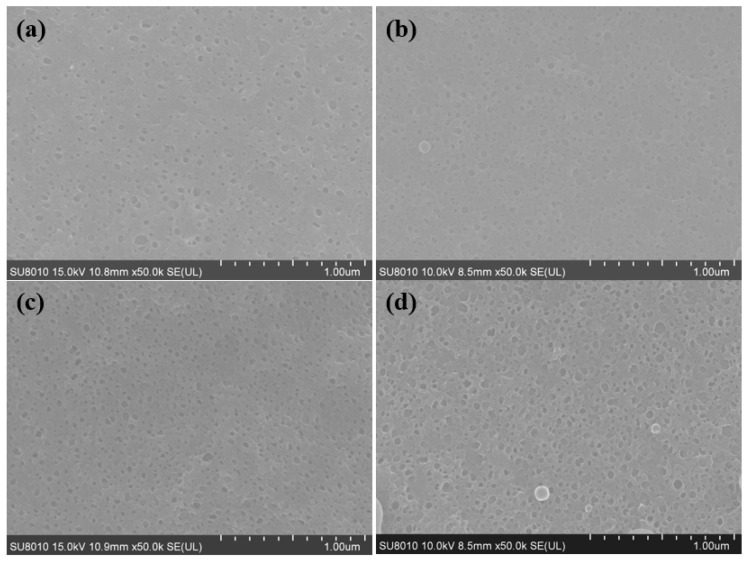

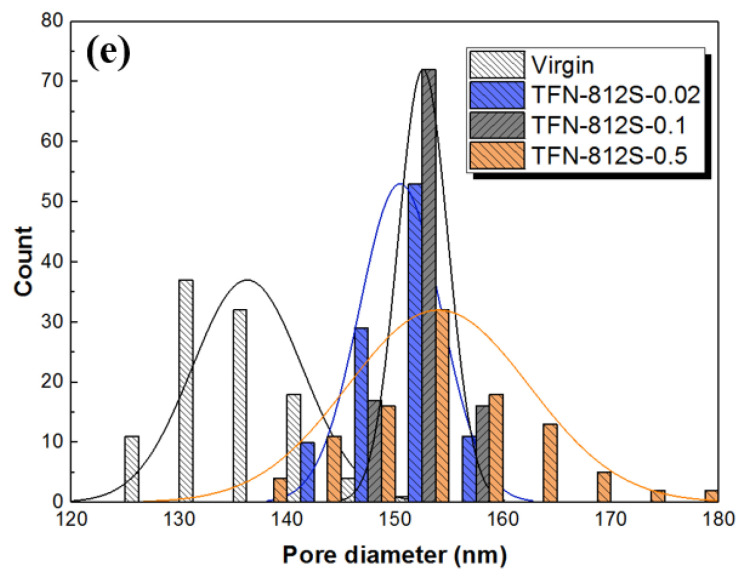
SEM images of the back surfaces of membranes with different concentrations of R812S NPs: (**a**) no NPs, (**b**) 0.02% (*w*/*v*), (**c**) 0.1% (*w*/*v*) and (**d**) 0.5% (*w*/*v*); (**e**) histograms of pore size in the back surfaces of membranes. The pore size distributions are generated based on 100 pore counts on the backside of the PA layer.

**Figure 7:**
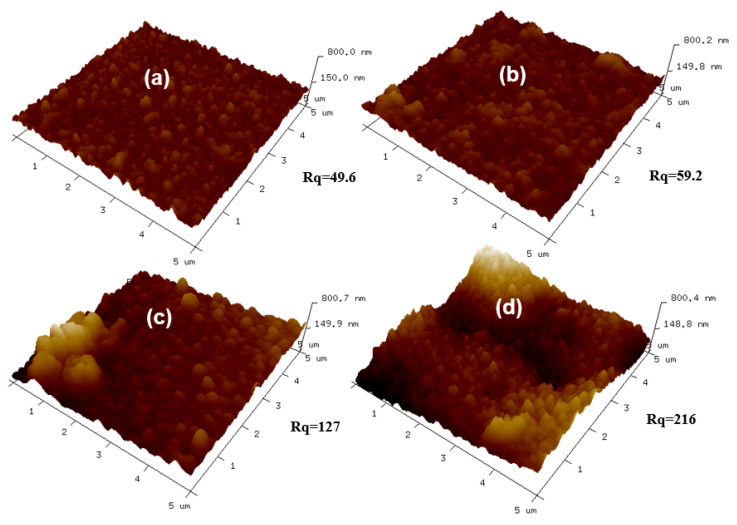
AFM images of the top surfaces of membranes with different concentrations of R812S NPs: (**a**) no NPs, (**b**) 0.02% (*w*/*v*), (**c**) 0.1% (*w*/*v*) and (**d**) 0.5% (*w*/*v*).

The morphologies of the pristine TFC and TFN membranes were observed by SEM, TEM, and AFM. The results are shown in Figure 4 (top surfaces observed via SEM and TEM), Figure 5 (cross-section observed via SEM and TEM), Figure 6 (back surfaces observed via SEM) and Figure 7 (top surfaces observed via AFM). The electron micrographs show that the surface of the unmodified membrane had a typical hybrid nodular and leaf-like morphology, which can be attributed to nanosized gas bubbles released during the IP reaction [29,35,36]. The TFC membrane features nodules with a size of around 193.6 ± 6.12 nm (Table 2 and Figure 4). Interestingly, as the addition of the R812S NPs, the size of the nodules decreased gradually to 145.2 ± 6.46 nm at 0.5% (*w*/*v*) concentration. Concurrently, the size and the frequency of the leaf-like structures increased significantly (Figure 4, left panel, inserted graph). As the leaf-like structures are shrunk large-sized nodules during hydration, this observation suggests that adding R812S NPs in the system promote the uneven distribution of the size of the nodules, which is likely due to high concentration R812S NPs inhibiting the diffusion of MPD molecules into the organic phase [24,37], thereby creating a more uneven morphology, featuring large-sized leaf-like structures and smaller nodules. Due to the appearance of the leaf-like structures, the TFN membrane has a much rougher membrane surface (for TFN-812S-0.5, *Ra* = 172 ± 10.3 nm) compared with the TFC membrane (*Ra* = 39.5 ± 8.7 nm). Note that when the addition of 812S NPs was 0.1% or larger, aggregated 812S NPs were found on the membrane surface due to aggregation of excess 812S NPs upon the removal of isopar-G solvent. These NPs may also contribute to enhanced roughness for TFN-812S-0.1 and TFN-812S-0.5 membranes. Generally, the rougher surface of the TFN membrane helps to increase the direct water contact area and therefore renders greater water flux [24,29,33,36]. Although larger higher NPs concentration may benefit the water flux, it should be cautioned that when the doping concentration exceeded 0.1% (*w*/*v*), NPs aggregates are frequently observed on the PA surface due to the agglomeration of NPs when the NPs are concentrated significantly as the organic solvent evaporates [38,39].

On the backside of the PA layer (Figure 6e), holes can be observed, which is in line with our previous findings [26,29,36]. The TFC membrane features holes with diameters mainly distributed at around 136.3 ± 5.11 nm, while the increase of R812S concentration generally enlarged the mostly distributed size to about 154 ± 2.41 nm for the TFN-812S-0.5 membrane. Moreover, with the increase of NPs concentration, the mean diameter of holes increased from 136 ± 5.11 to 154 ± 2.41 nm. This increased trend corresponds well with the appearance of leaf-like structures on the front surface, as the large-sized structures are formed through larger holes on the back surface by the more intense release of gas nanobubbles [29,35,36]. The enhanced size and density of such holes could efficiently increase the flux of the TFN-RO membranes, considering these holes connect the voids in the polyamide layer with the pores on the PSF substrate [29,36].

Although the appearance of the leaf-like structures increased the thickness at some location, the average apparent thickness (Table 2) of the PA layer decreased from 124 ± 7.2 nm to 109 ± 8.1 nm as 0.5% (*w*/*v*) R812S NPs was added. On the contrary, the intrinsic thickness (namely, the thickness of the polyamide nanofilm that forms the walls of the nodules) increased steadily from 19.1 ± 1.06 nm to 22.4 ± 0.06 nm as the concentration of R812S NPs increased from 0% to 0.5% (*w*/*v*). This phenomenon can be explained by the addition of R812S NPs in the organic phase inhibit penetration of MPD molecules further into the organic phase, therefore generating a limiting growth mechanism, which decreases the apparent thickness [37]. Rather, limited MPD molecules accumulated at the near interface, increasing the local MPD concentration, therefore accelerating the interfacial polymerization reaction at the location, forming denser and thicker intrinsic PA nanofilms. According to the analysis in the previous publication, the denser and thicker PA nanofilm is more favorable for the salt rejection, but slightly decreases water permeability [29].

### 3.3. Compositions of the Membranes

**Figure 8:**
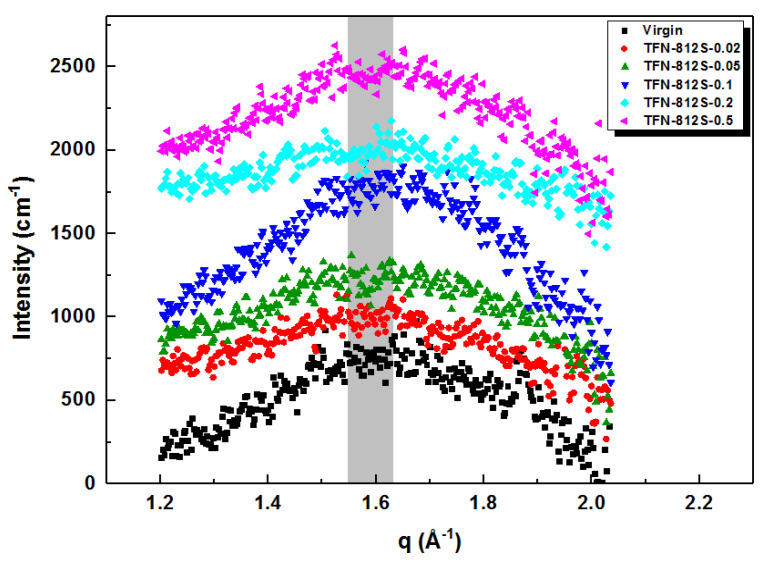
SAXS spectra of membranes with different concentrations of R812S NPs.

SAXS data were obtained by the relation between d and q defined by the Bragg equation: 2dsinθ = λ and q = 4πsinθ/λ, where d represents the pore size of the membrane surface and q represents the position of the scattering peak. Figure 8 shows that the q peaks were at approximately 1.6 Å^−1^, which corresponds to an average chain distance of 0.39 nm according to the Bragg equation. Here, the average chain distance did not show much change as to increasing the doping amount of the alkyl-silica NPs. Besides, the gyration radius and unit structure radius of the membranes (Table 3) reflects the stacking size of clusters of polymer and primary particle’s molecular weight, respectively [40,41,42,43]. The gyration radius decreased first with a small amount of addition and then gradually increased as more NPs were added. Meanwhile, the primary unit radius showed no obvious trend, suggesting that the molecular weight of the primary unit was not impacted by the doping [38]. The SAXS suggests that the stacking behavior of the primary clusters is affected by the doping of silica NPs, suggesting the successful doping of NPs in the polymer network. However, the primary unit radius and the average chain distance showed no obvious change by the doping.

**Figure 9:**
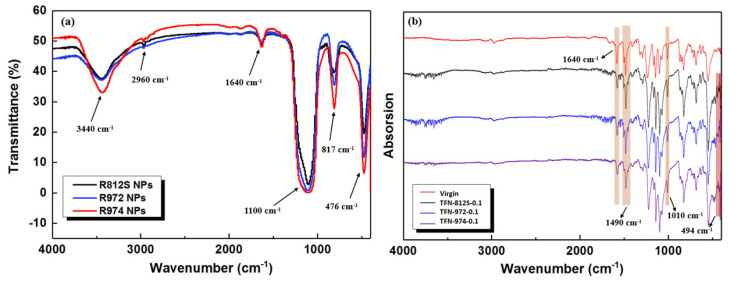
(**a**) FT-IR spectra of the different types of NPs and (**b**) ATR-FTIR spectra of the TFN membranes containing different NPs at the concentration of 0.1% (*w*/*v*).

Figure 9a shows that the FTIR spectra of the different alkyl-silica NPs used in this study were generally similar. This transmittance band centered at 3440 cm^−1^ was assigned to the stretching vibration of (Si‒) O‒H, which is probably the residue –OH groups not been salinized [26,44]. Characteristic methyl peaks appeared around 2960 cm^−1^. The weak peak near 1640 cm^−1^ was attributed to the H‒OH bending/stretching vibration of water. In the spectra of the NP-doped membranes, bands were observed at 1100, 817, and 476 cm^−1^, which corresponds to the vibration absorption of Si‒O‒Si groups [45,46].

The infrared spectra shown in Figure 9b shows the comparison between ATR-FTIR spectra of the TFN membranes containing different NPs at the concentration of 0.1% (*w*/*v*). They all exhibit large bands centered at 1660, 1490, and 1580 cm^−1^, which correspond to the C=O bond of amide I band, stretching vibration of the hydrogen bond-forming amide II and the plane bending vibration of the N‒H bond of amide II band [33], respectively. The appearance of these characteristic peaks suggests that the IP of the MPD aqueous phase and TMC organic phase resulted in a PA separation layer, as expected. Furthermore, the characteristic peaks at 1010 and 494 cm^−1^ correspond to Si‒O‒Si anti-symmetric stretching vibration absorption and Si‒O symmetric stretching vibration absorption, indicating that the alkyl-silica NPs were successfully doped into the membranes [15,26,47].

### 3.4. Surface Properties of the Membranes

**Figure 10:**
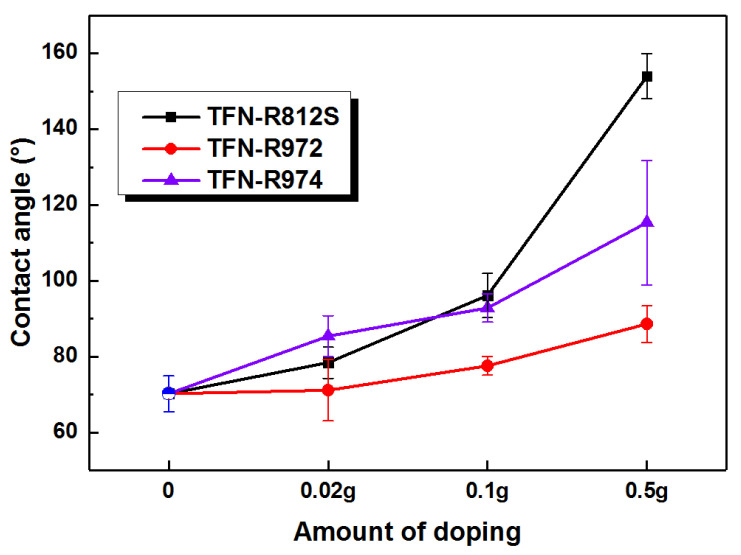
Contact angles of the membranes with different concentrations of NPs.

The hydrophilicity, commonly evaluated by measuring the contact angle, reflects how the membrane surface interacts with water molecules. Generally, the more hydrophilic a membrane surface is, the higher the permeation flux of the membrane will be. For example, Zhao et al. showed that the addition hydrophilic o-ABA–TEA to the MPD aqueous solution yields RO membranes with higher flux [11]. However, we found that hydrophobic alkyl groups capped SiO_2_-NPs, although they resulted in a more hydrophobic membrane surface, also enhancing the flux. As shown in Figure 10, increasing the concentration of NPs increased the contact angle (i.e., made the membrane more hydrophobic) compared to the TFC-RO membrane. This finding implies that the successful incorporation of the hydrophobic NPs during the IP process [8] and that the hydrophobic end methyl groups render the membrane surface more hydrophobic. When the concentration of R812S NPs was greater than 0.1% (*w*/*v*), the contact angle increased sharply, which may be associated with the aggregated superhydrophobic alkyl-silica NPs which partially covered the membrane surface (Figure 4e,f). On the contrary, the increases associated with high concentrations of R972 and R974 NPs were more moderate, which may suggest less accumulation of R972 and R974 NPs on the membrane surface upon solvent drying. The impact of the R972 NPs on the contact angle was smaller than that of the R974 NPs, which was probably related with the difference between their specific surface areas. As shown in Table 4, according to Wenzel’s equation, the real contact area of the solid–liquid interface on the rough surface is larger than the apparent contact area. The apparent contact angle θ* of a rough surface can be computed using the Young’s equation [40]. Where r is the roughness factor (that is, SAD value), for the original hydrophilic surface (**θ** < 90°), the larger the r value is, the smaller the θ*. For hydrophobic surface (**θ** > 90°), the larger the r value, the greater the θ*. That is, surface roughness can make the hydrophilic surface more hydrophilic and the hydrophobic surface more hydrophobic.

**Figure 11:**
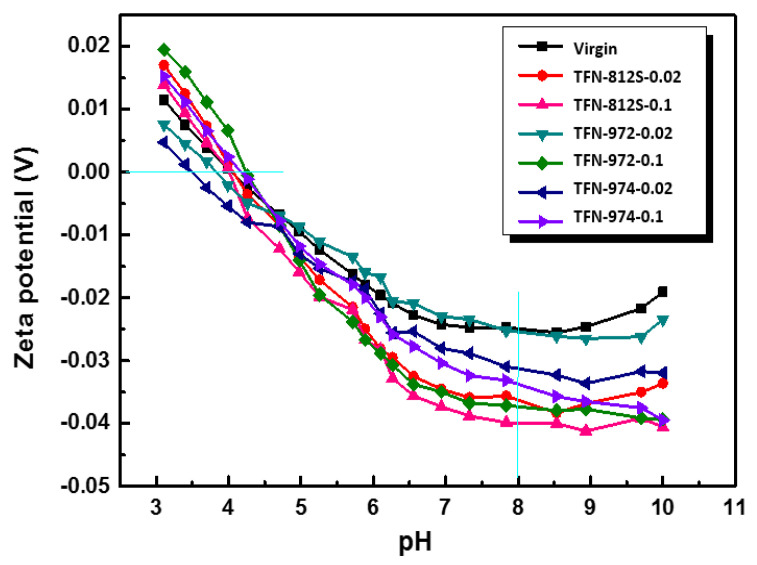
Zeta potentials of the membranes with different concentrations and types of NPs.

Next, the Zeta potential of each membrane surface was measured as a function of the pH (Figure 11). The surface charges of the membranes with and without NPs became negative around pH = 4.2 and continued decreasing as the pH increased further it reached 7.8 (this was the pH range used for performance testing). This change in the surface charge was mainly due to the dissociation of the carboxyl and protonation of amine groups. It can be generally observed that as the increment of the doped alkyl-silica NPs, the Zeta potential was decreased, suggesting the presence of more carboxylic groups on the membrane surface. This phenomenon can be explained considering the “confining effect” of the hydrophobic alkyl-silica NPs. As a higher concentration of NPs was applied, the MPD diffusion is more significantly limited to near the surface, thereby creating a denser PA layer at the backside of the PA layer. Meanwhile, as the MPD diffusion to the TMC is inhibited, the PA front surface has a lower MPD/TMC ratio, resulting in a less dense PA layer, featuring more dissociated carboxylic groups. This phenomenon agrees well with the FESEM observation that as the NPs concentration increases, the apparent thickness of the PA layer was decreased while the intrinsic thickness of the PA layer was enhanced.

### 3.5. Demonstration of Separation Performance

The performance of each RO membrane was evaluated in terms of the pure water permeability coefficient (*A*), solute permeability coefficient (*B*), water flux (*J*), and solute rejection (*R*). In this experiment, the permeability flux and salt rejection of all membranes were measured for DI water, 2000, and 32,000 ppm NaCl solution (simulating brackish water and seawater) in sequence. The *A* and *B* values were calculated from *J*, *R,* and the experimental parameters (temperature, operating pressure, and salinity).

**Figure 12:**
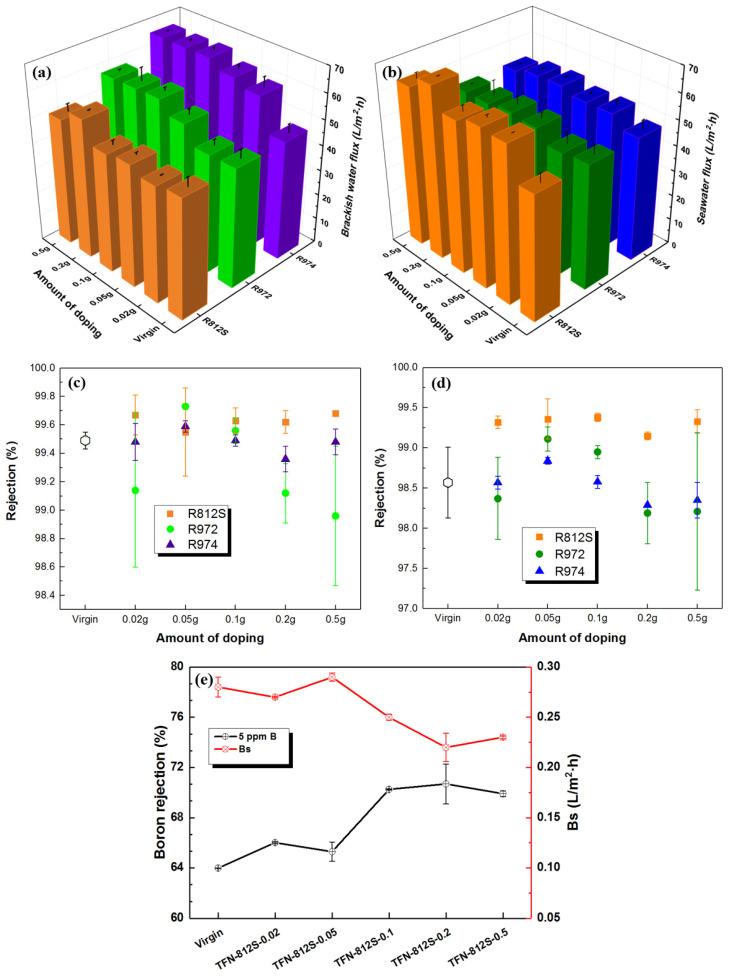
The measured flux of (**a**) brackish water, (**b**) seawater and salt rejection of (**c**) brackish water, (**d**) seawater of membranes doped with three kinds of NPs at different concentrations; (**e**) boron rejection of membranes doped with different concentrations of R812S NPs.

As shown in Figure 12a, with the simulated brackish water, the flux initially was promoted with increasing NPs concentration and then decreased at higher NPs concentrations. At the optimal NPs concentration of 0.1% (*w*/*v*), the maximum flux of the membrane doped with R812S NPs was 55.3 L/m^2^∙h, while those doped with R972 and R974 NPs were 58.02 and 65.84 L/m^2^∙h, respectively. Compared with the TFC membrane, TFN-812S, TFN-972, and TFN-974 membranes increased the water flux by 8.04, 10.66, and 18.48 L/m^2^∙h, respectively. Similarly for seawater, the permeation flux (Figure 12b) was improved due to the incorporation of hydrophobic alkyl-silica NPs. In comparison with the membrane without NPs, the flux increased by 18.7, 4.5, and 6.12 L/m^2^∙h with optimum NPs doping.

Generally, there should be two mechanisms that govern the water permeability: the hydrophobicity of the membrane surface, which generally decreases the membrane flux; and the surface roughness, which enhances the membrane flux. The more hydrophobic property of the R812S-modified TFN-RO membranes may explain the less increase at low-pressure brackish conditions. However, at higher operation pressure seawater desalination conditions, the effect of the hydrophobicity became minor and the enhancement of roughness became major, therefore the R812S-modified TFN-RO membrane exhibited the highest flux increment.

Depending on the doped concentration of alkyl-silica NPs, the salt rejection was maintained, or even slightly increased, compared to the unmodified membrane at the brackish water condition. At optimum doping conditions, the rejection rate achieved was 99.68%, 99.73%, and 99.59%, compared with the virgin membrane (99.49%). Similarly, at seawater conditions, the salt rejection reached approximately 99.38%, 99.11%, and 98.84% at the optimal R812S, R972, and R974 NPs concentrations, respectively. The TFN-812S RO membranes led to better boron removal of 11% improvement compared with the virgin membrane. The enhancement of the salt rejection can be explained by a net result of higher intrinsic thickness, hydrophobic plugging, and higher surface charge. While the higher rejection of boron should be attributed to the higher intrinsic thickness and the hydrophobic plugging effect.

### 3.6. Performance Comparison between the TFN Membranes in This Work and RO Membranes in the Literature

**Figure 13:**
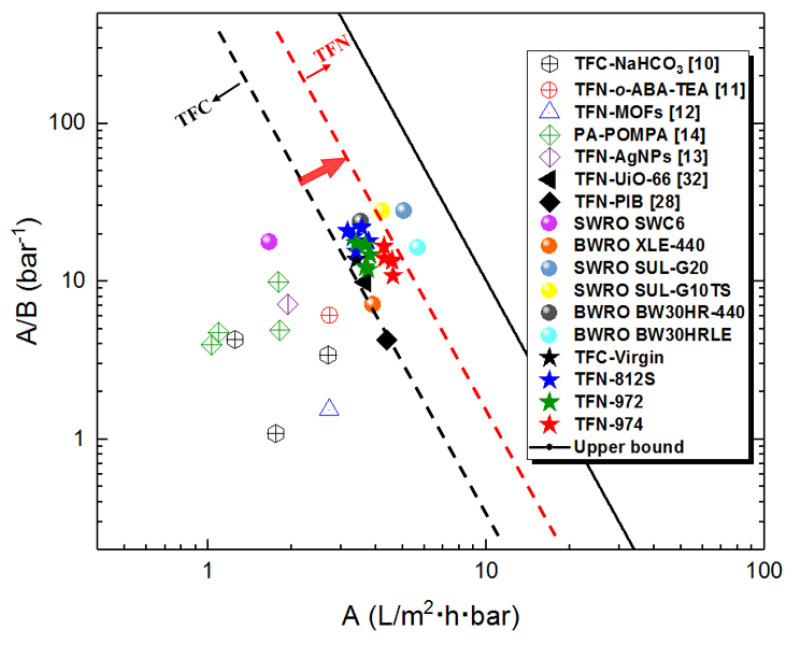
The performance comparison of RO membranes in this work with other RO membranes reported in the literature (including the commercial membranes in the reports). All membranes were tested using 2000 mg L^−1^ NaCl feed solution under 16-bar pressure. While TFN-MOFs, TFN-AgNPs, TFN-UiO-66, and TFN-PIB were under 15.5 bar, and PA-POMPA was under 15 bar. TFN-o-ABA-TEA was tested at 32,000 mg L^−1^ NaCl and operating pressure of 55 bar. The upper bound was based on the water/NaCl selectivity A/B and water permeability coefficient A for TFC polyamide membranes [48].

The desalination properties of RO membranes prepared in this work are compared with other RO membranes reported in the literature (including the commercial membranes in the reports). As shown in the permeability–selectivity tradeoff graph (Figure 13), membranes in this study display a superior performance compared to the other TFN RO membranes in the literature [10,11,12,13,14,28,32], and even match the performance (for the TFN-812S-0.5 membrane) of a few SWRO (seawater reverse osmosis) commercial membranes. The TFN membranes developed in this work are closer to the upper bound, and their separation performance is further improved than the control TFC membrane. This is because the high concentration alkyl-silica NPs in organic solvent have shaped the nanoscale structure and separation properties of the PA layer with high performance.

## 4. Conclusions

Herein, aromatic polyamide TFN-RO membranes were fabricated by incorporating hydrophobic alkyl-silica NPs with the organic solution, which experienced an IP reaction with the MPD aqueous solution to form an imbedded nanocomposite polyamide layer. The TFN-RO membranes were characterized in detail by SEM, TEM, AFM, SAXS, FTIR, contact angle measurements, and Zeta potential measurements, and the results confirmed the successful doping of NPs in the membranes. In addition, we successfully demonstrated the modification of alkyl groups is very effective to reduce the agglomeration of NPs in the membranes and greatly enhanced the properties of the membranes. Foremost, the confined growth of PA layer by providing diffusion resistance by the high concentration alkyl-silica NPs resulted in a rougher surface and higher intrinsic thickness, which increased permeability, salt rejection of the TFN-RO membranes. Secondly, the hydrophobic methyl groups render a hydrophobic plugging effect to the polyamide network, resulting in less hydrogen bonding and less polar PA surface and therefore induced a hydrophobic plugging effect, which increased the boron rejection. As a net result, the TFN-RO membranes showed improved water permeability and higher salt rejection at both brackish and seawater desalination conditions. This approach can provide a novel and facile approach to producing a high-performance TFN-RO membrane with good upscale potential. Besides, as the alkyl group has low surface energy, which reduces the initial attachment of the foulants, which are typically polar, on the membrane surface. Hence, it is possible that such properties could also bring about anti-fouling properties. As the current work focused mainly on the fabrication, characterization, and mechanistic studies of the flux and rejection enhancement by incorporating the alkyl group capped silica NPs in the organic phase during IP. There are some related topics, such as anti-fouling properties by the low-energy alkyl group and long-term stability of the resultant TFN membrane. These issues will need to be systematically studied in the following work.

## Figures and Tables

**Table 1:** Basic parameters of the commercial hydrophobic NPs, as reported by the manufacturers.

NPs Types	Preparation (Compositions)	Specific Surface Area (BET) (m^2^/g)	C Content (%)
R812S	fumed silica treated with HMDS ^a^	195–245	3.0–4.0
R972	fumed silica treated with DDS ^b^	90–130	0.6–1.2
R974	fumed silica treated with DDS ^b^	150–190	0.7–1.3

^a^ Hexamethyldisilazane, ^b^ Dimethyldichlorosilane.

**Table 2:** Data comparison of the TFC and TFN RO membranes.

Membrane	Intrinsic Thickness ^a^ (nm)	Apparent Thickness ^b^ (nm)	Hole Size of Back Surface ^c^ (nm)	Diameter of Nodules ^d^ (nm)	*A*^e^ (LMH/bar)	*A*/*B*^f^ (bar^−1^)
Virgin	19.1 ± 1.06	124 ± 7.2	136.3 ± 5.11	193.6 ± 6.12	3.42	13.71
TFN-812S-0.02	19.5 ± 0.67	120 ± 8.7	150.4 ± 3.78	179.4 ± 9.09	3.17	19.12
TFN-812S-0.1	22.0 ± 1.02	113 ± 5.0	152.6 ± 2.32	154.9 ± 5.23	3.32	20.84
TFN-812S-0.5	22.4 ± 0.06	109 ± 8.1	154.0 ± 2.41	145.2 ± 6.46	3.57	21.94

^a^ Based on 10 measurements in TEM images of cross-sections of membranes; ^b^ Based on 10 measurements in SEM images of cross-sections of membranes; ^c^ Based on 100 measurements in SEM images of back surfaces of membranes; ^d^ Based on 50 measurements in TEM images of top surfaces of membranes; ^e^ Based on the pure water permeability coefficient; ^f^ Based on the water/NaCl selectivity.

**Table 3:** SAXS comparison of membranes with different concentrations of R812S NPs.

Membrane	Gyration Radius (nm)	Primary Structure Radius (nm)	Chain Distance (nm)
Virgin	25.2	18.6	0.39
TFN-812S-0.02	24.4	17.8	0.39
TFN-812S-0.1	25.0	18.3	0.39
TFN-812S-0.2	26.5	19.4	0.39
TFN-812S-0.5	26.3	18.5	0.39

**Table 4:** Surface properties of the TFC and TFN RO membranes.

Membrane	*R_a_* (nm)	*SAD*^a^ (%)	Contact Angle ^b^ (°)	θ* ^c^ (°)	Zeta Potential ^d^ (mV)
Virgin	39.5 ± 8.7	43.2 ± 2.9	70.2 ± 5.0	81.4 ± 2.1	−24.7
TFN-812S-0.02	46.3 ± 9.1	46.0 ± 2.6	78.5 ± 3.8	84.8 ± 2.1	−36.1
TFN-812S-0.1	90.9 ± 11.6	66.3 ± 4.3	96.2 ± 5.6	94.0 ± 5.6	−40.0
TFN-812S-0.5	172.0 ± 10.3	80.5 ± 5.5	153.9 ± 7.1	136.1 ± 11.0	−41.5

^a^ Based on the surface area difference in AFM images of roughness results of membranes; ^b^ Based on six measurements in the static water contact angle of top surfaces of membranes; ^c^ Based on the Wenzel’s equation cosθ* = *r* cosθ [40], where the roughness factor, r, can be measured as surface area difference (*SAD)* in AFM analysis.; ^d^ pH = 7.8 during the membrane performance test.
